# Opinions and attitudes toward artificial intelligence among operating room nurses: a descriptive meta-analysis based on the comparative studies of the different opinions

**DOI:** 10.3389/frai.2025.1681994

**Published:** 2025-11-19

**Authors:** Juhua Ye, Junwu Huang

**Affiliations:** 1 Department of Anesthesiology, Huanggang Central Hospital of Yangtze University, Huanggang, Hubei, China; 2 Department of Orthopaedics, Huanggang Central Hospital of Yangtze University, Huanggang, Hubei, China

**Keywords:** operating room nurse, artificial intelligence, opinions, robotic nursing, patient care

## Abstract

**Background:**

Artificial intelligence (AI) is defined as the capability of machines to perform tasks that typically require human intelligence. Robots have major roles during surgeries as well as in the operating rooms (ORs). Therefore, it is expected for nurses working in ORs to be knowledgeable about those new technologies and the preparation of robots for surgeries. In this analysis, we aimed to represent the opinions and attitudes of ORs nurses toward AI.

**Methods:**

Online databases were searched for relevant publications. AI based questions were asked to the ORs nurses and the percentage of participants who agreed or disagreed to specific questions were recorded. The RevMan application was used to carry out statistical analysis, whereby odds ratios (OR) and 95% confidence intervals (CI) were used to represent the results.

**Results:**

Six studies consisting of a total number of 1,197 participants were included. ORs nurses believe that AI and robotic nursing applications will significantly reduce the workload of nurses with OR: 75.73, 95% CI: 8.28–692.86; *p* = 0.0001. In addition, a majority of ORs nurses significantly accepted the application of AI in nursing (OR: 63.70, 95% CI: 2.15–1890.57; *p* = 0.02) and significantly believed that AI will revolutionize in the field of nursing (OR: 15.27, 95% CI: 3.47–67.15; *p* = 0.0003). In addition, they significantly agreed that robotic technologies are very important (OR: 12.57, 95% CI: 6.44–24.54; *p* = 0.00001). The ORs nurses significantly disagreed to the fact that robotic technologies are too expensive and unnecessary (OR: 0.02, 95% CI: 0.01–0.04; *p* = 0.00001). Nevertheless, even though majority of the ORs nurses agree that robotic checking system is time consuming, the result was not significant (OR: 1.38, 95% CI: 0.79–2.40; *p* = 0.26).

**Conclusion:**

Majority of the nurses believe that AI and robotic nursing applications will significantly reduce the workload of nurses, they believe that AI will significantly revolutionize in the field of nursing, and they believe that robotic technologies are very important. However, due to the several limitations from this analysis, the results should be considered with caution.

## Introduction

Artificial intelligence is defined as the capability of machines to perform tasks that typically require human intelligence (Bottacin et al., 2025). This ability for a machine to think and learn and its combination with advances in computational power and data storage and the easily available large high quality digital data sets and machine learning frameworks have rendered scientists to further explore more research on artificial intelligence, especially in the health care sector.

Artificial intelligence in the health care sector could likely increase decision making, help in the effective management of patients, and could predict health outcomes, accelerate diagnosis and could more efficiently screen patients for emergencies and specific diseases (Alali et al., 2025; Hsu et al., 2025; Cho et al., 2025; Liu et al., 2025; Marchi et al., 2025). Several artificial intelligence platforms have already found a place in surgery and surgical procedures (Nishiya et al., 2024; Isch et al., 2025). Artificial intelligence might now be used to improve decision making pre and post operatively, in pre-operative diagnosis, predicting post-operative outcomes and reporting, as well as in surgical planning. Artificial intelligence can be used in different types of surgeries including neurosurgery and vascular surgeries (Kono, 2025).

Today, artificial intelligence in operating room is also gaining attention. Operating rooms, where surgical interventions are carried out, have a multidisciplinary work team including the anesthesiologists, the surgeons, the operation room nurses, the technicians and the pharmacists. Nowadays, patients are being served with new approaches thanks to artificial intelligence (Mindy Duffourc et al., 2025). Robotic technologies integrated with artificial intelligence are gradually making their entry in operating rooms and surgeries. This new technology called robotic-assisted surgery is gaining more attention and becoming more attractive in this new era of artificial intelligence (Moschovas et al., 2025).

Robots have major roles during surgeries as well as in the operating rooms. Therefore, it is expected for nurses working in operating rooms to be knowledgeable about those new technologies and the preparation of robots for surgeries. Hence, operating room nurses should improve themselves and keep them up to date in order to adapt and work with new technologies driven by artificial intelligence.

Artificial intelligence will rule the world in the near future. Several new studies based on artificial intelligence have recently been published. Studies based on the implementation of Artificial intelligence in operation room and surgeries are gradually showing their impact. However, up to now, no meta-analysis or systematic review or descriptive analysis has shown the opinions and attitudes of operating room nurses toward Artificial intelligence. Such an analysis might show the viewpoints and opinions of the operating room nurses about artificial intelligence in the operation room and robotic surgery. It is important to analyze the opinions and views of the operating room nurses to understand whether they can adapt to this new working environment.

Few studies have been published based on the opinions and attitudes of operating room nurses toward artificial intelligence and robotic surgeries. However, conclusions from individual studies are often controversial. Therefore, through pooled data from several studies, the effective sample size could increase, as well as the consistency of results across studies could better be assessed thereby generating a more efficient overall estimate of the effect, with higher level of evidence that could be used to develop clinical practice guidelines and inform decision making. Therefore, because of the several positive aspects of pooled analyses compared to a single individual study, we aimed to represent the opinions and attitudes of operating room nurses toward artificial intelligence through this analysis.

## Methods

### Search databases

Search databases including MEDLINE, EMBASE, Google scholar, ClinicalTrials.gov, the Cochrane databases and Web of Science were searched for relevant publications showing the opinions and attitudes of operating room nurses toward artificial intelligence.

### Search strategies

During this search process, the following searched terms were used:

- ‘nurses and artificial intelligence’;
- ‘operating room nurses and artificial intelligence’;
- ‘nurses and robotic surgery’;
- ‘operating room nurses and robotic surgery’;
- ‘nurses and operating room and artificial intelligence’;
- ‘nurses and operating room and robotic nurses’.

References of the relevant studies were also checked for suitable research papers to be included in this analysis.

### Criteria for inclusion and exclusion

Inclusion criteria were:

- Studies that were based on opinions and attitudes of operating room nurses toward artificial intelligence.

Criteria for exclusion were:

- Literature reviews, systematic reviews or meta-analyses;
- Case studies;
- Editorials or commentaries;
- Repeated studies.

A PICOS (P = Population; I = Intervention; C = Comparison; O = Outcome; S = Study type) approach to this study has been represented in Table 1.

**Table 1:** Represents a PICOS approach to this study.

A PICOS approach in this study	
P = Population	A population of operating room nurses
I = Intervention	Questions based on the opinions and attitudes about operating use nurses
C = Comparison	Comparing the opinions and attitudes about operating room nurses
O = Outcome	Response to the questions based on the opinions and attitudes about operating room nurses
S = Study Type	Any study type except meta-analyses, systematic reviews, literature reviews, case studies, commentaries and editorials

PICOS (P = Population; I = Intervention; C = Comparison; O = Outcome; S = Study type).

### Questions which were asked to the operation room nurses

Table 2 showed questions based on artificial intelligence which were asked to the operating room nurses and the percentage of participants who agreed or disagreed to specific questions from the different original studies.

**Table 2:** Questions which have been asked to the nursing staffs.

Studies	Questions	Yes	No
Ergin et al. (2023)	Have you ever heard of the concepts of artificial intelligence (AI) and robotic nursing?	75.4%	23.7%
	Do you think robots with AI will replace nurses?	28.6%	71.4%
	Do you think robots with AI will benefit the nursing profession?	77.1%	22.9%
	Do you think AI and robotic nursing applications will reduce the workload of nurses?	80.0%	20.0%
Horsfall et al. (2021)	Do you agree with the use of an AI system in pre-operative imaging interpretation?	Majority somewhat agreed	–
	Do you agree with the use of an AI system in surgical team coordination?	Majority somewhat agreed	–
	Do you agree with the use of an AI system in Operative planning?	Majority somewhat agreed	–
	Do you agree with the use of an AI system in intra-operative safety alerts?	Majority strongly agreed	–
	Do you agree with the use of an AI system in Robotic surgery?	Majority somewhat agreed	–
	Do you agree with the use of an AI system in post-operative management?	Majority somewhat agreed	–
Karaarslan et al. (2024)	Can robots with AI replace nurses?	27.9%	72.1%
	Can AI and robotic nurses reduce nurses’ workload?	83.7%	16.3%
	Would you be willing to use AI based technologies?	60.5%	39.5%
	Would you like to work with robot nurses in your professional life?	58.2%	11.6%
	What feeling does AI create in you?		
	Do you fear that AI will create ethical health related problem in the future?	46.5%	16.3%
	Is provision of health care by a robot nurse safe?	23.3%	25.5%
	Does the use of AI and robot nurses in healthcare increase patients’ satisfaction?	27.9%	27.9%
	Does the use of AI and robot nurses in healthcare increase efficiency of healthcare?	44.2%	16.3%
Porto and Catal (2021)	Are robotic technologies complicated?	13.8%	86.2%
	Does robotic technology help to provide a safer care?	82.1%	17.9%
	Do you think robotic technology bring a significant change?	89.4%	10.6%
	Do you think robotic technologies are too expensive and unnecessary?	12.1%	87.9%
	Do you think robotic technologies reduce surgery risks?	80.6%	19.4%
	Do you think robotic technologies are very important?	77.6%	22.4%
	Do you think being part of the robotic technology makes oneself proud?	78.1%	21.9%
	Do you think robotic technologies work appropriately?	0%	100%
	Do you think robotic checking system is time consuming?	53.8%	46.2%
	Do you think that patient positioning in robotic surgery is difficult?	27.7%	72.3%
	Do you think technical support for the Da Vinci robotic system is insufficient?	24.2%	75.8%
Wang et al. (2024)	What do you think of the current development of AI in nursing?	23.8%	2.4%
	Do you understand the application of AI in nursing?	21.4%	11.9%
	Do you understand AI?	32.0%	7.7%
	Do you agree that AI will revolutionize the field of nursing?	79.7%	20.3%
	Do you agree that application of AI in nursing can improve patients care?	92.6%	7.5%
	Do you agree that the application of AI in nursing can improve nursing decision taking?	86.5%	13.5%
	Do you agree that AI in nursing can improve the health of population?	87.7%	12.2%
	Do you agree that AI in nursing can reduce healthcare costs?	84.9%	15.1%
	Do you agree that AI in nursing can reduce the burden on healthcare workers?	93.9%	6.0%
	Do you agree that AI in nursing will change the role of nurses in the future?	82.8%	17.2%
	Do you agree that AI in nursing will replace the work of nurses?	57.2%	42.9%
	Do you accept the application of AI in nursing?	97.9%	2.1%

Based on the percentage of nurses who agreed and disagreed to the questions which were asked (Table 2), an average mean which was reported as percentage as well as a statistical analysis was carried out.

### Data extraction, quality assessment and statistical analysis

The authors carefully read all the selected studies and independently extracted data from those questionnaire surveys. The percentages and number of participants who agreed or disagreed with the questions interviewed were collected and tabulated. However, before this step, the authors’ names were extracted as well as the year of publication, the time period of participants’ enrolment as well as the type of study. The country of original from where these participants were enrolled as well as data related to the type of nurses included in this research were also extracted. Further, the total number of participant nurses, the year of conduction of the research, the country involved, and the type of studies were all extracted and tabulated. Data concerning the methodological quality were also collected.

During this search process, any disagreement or any unclear data inclusion was carefully discussed among the authors and then clarified.

The quality of the studies based on a methodological assessment was carried out by the Newcastle Ottawa Scale (NOS) where stars were given to judge the bias risk (Margulis et al., 2014).

The NOS was used to assess bias risk for the non-randomized trials. This tool was developed to assess the quality of non-randomized studies based on its design, content and ease of use directed to the task of incorporating the quality assessments in the interpretation of the descriptive analytic results. A ‘star system’ has been developed in which a study is judged on three broad perspectives: the selection of the study groups; the comparability of the groups; and the ascertainment of either the exposure or outcome of interest for case–control or cohort studies respectively:

(a) Selection:

- Representative of the exposed cohort;
- Selection of the external control;
- Ascertainment of exposure;
- Outcome of interest not present at the start of the study.

(b) Comparability:

- Main factor and additional factor based on comparability of cohorts.

(c) Outcome:

- Assessment of the outcomes;
- Sufficient follow-up time period;
- Adequacy of follow-up.

This analysis used data answered through survey questionnaires. Questions related to artificial intelligence were asked to the nurses, and based on their agreement or disagreement given in their answers, a mean percentage was derived.

The mean percentages of participants who agreed or disagreed to specific artificial intelligence based questions were reported as results. Based on different questionnaires, common questions were selected and a percentage of the response was represented in the form of results.

In addition, statistical analysis was carried out by the Review manager software (version 5.4). During analysis, the percentage of participants who agreed to specific questions and the percentage of participants who did not agree to specific questions were filled up as data in the Revman application. Since the total number of participants was not reported in the original studies, we have better used a sample of 100 participants in each group just for comparison. To better illustrate this, we have given an example. Suppose 27.8% of the participants agreed with the use of artificial intelligence in the operating room, and 72.2% did not agree to the use of artificial intelligence in the operating room, we have considered the number of participants who agreed to use artificial intelligence as *n* = 28, among a total number of 100 participants, and the number of participants who did not agree for the use of artificial intelligence as *n* = 72, among a total sample size of 100 and we have filled up this information in the Revman application in order to generate a result.

Heterogeneity was assessed using the I^2^ statistic test as well as the Q statistic test. A subgroup analysis which generated a *p* value less or equal to 0.05 was considered statistically significant whereas a subgroup analysis which generated a *p* value above 0.05 was considered insignificant statistically. Based on the I^2^ statistic test, heterogeneity increased with an increasing I^2^ value. In our analysis, a random effects statistical model was used during data analysis.

Odds Ratios (OR) with 95% confidence intervals (CI) were used to represent the statistical analysis.

### Ethical approval

This article is based on previously conducted studies and does not contain any new study with human participants or animals performed by any of the authors.

## Results

### Search outcomes

A total number of 68 publications were obtained through the search process. Because this study was a descriptive analysis, we decided to use the preferred reporting items in systematic reviews and meta-analyses (PRISMA) guideline (Page et al., 2021) during this search process.

After carefully assessing the titles, abstracts as well as the full texts of the 68 publications, studies which were not relevant to this analysis were directly eliminated remaining with only the eligible studies. Full text articles were eliminated for the following reasons: literature review (1), case study (2) and duplicated studies (12).

**Figure 1:**
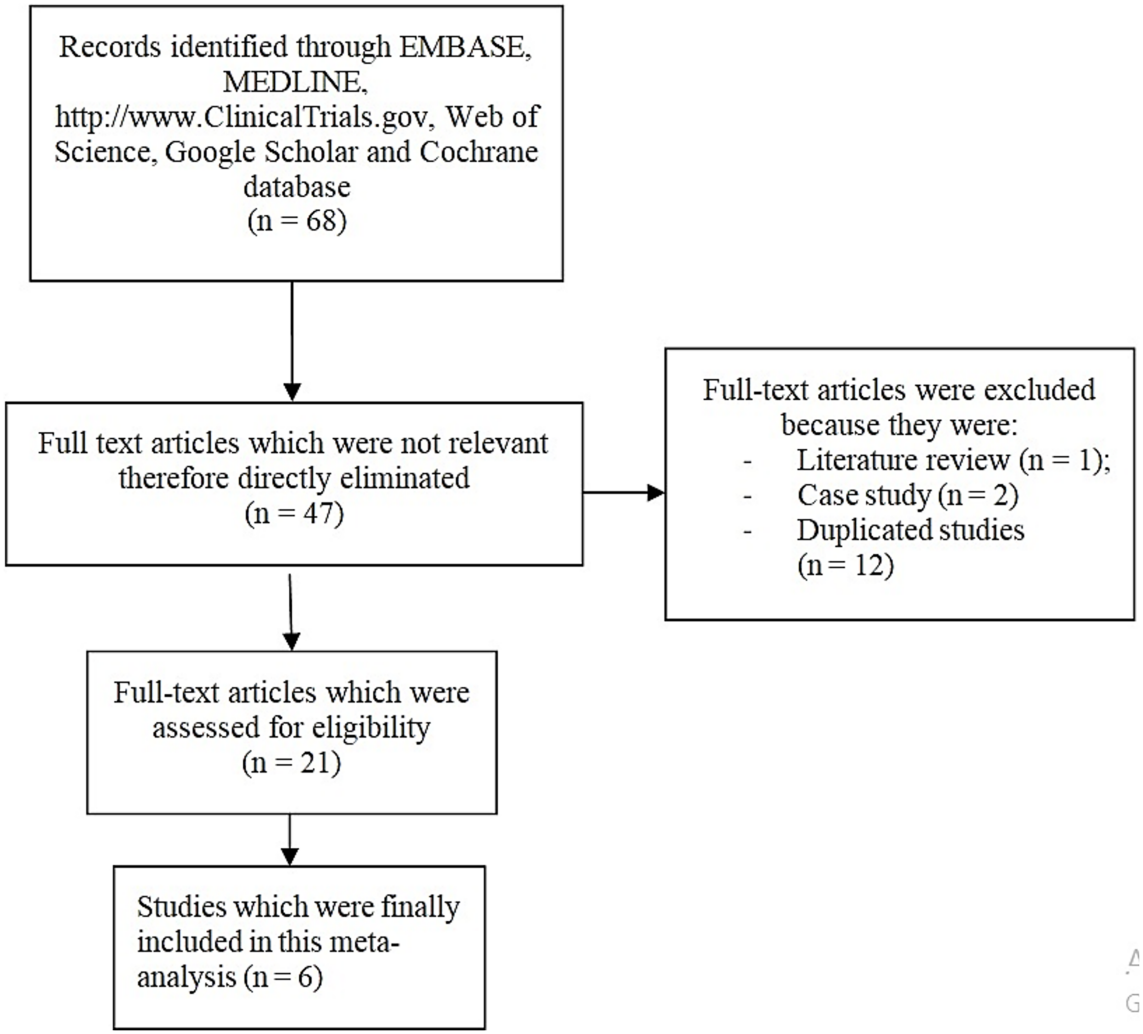
Flow diagram showing the study selection. The PRISMA guideline was followed during the search process. Following this search, a total number of 68 publications were obtained. After carefully assessing the titles, abstracts as well as the full texts of the 68 publications, studies which were not relevant to this analysis were directly eliminated remaining with only the eligible studies. Full text articles were eliminated for the following reasons: literature review (1), case study (2) and duplicated studies (12). Following the elimination of duplicates and eligibility criteria check, only 6 studies were finally selected for this analysis.

Following the elimination of duplicates and eligibility criteria check, only 6 studies () were finally selected for this analysis as shown in Figure 1.

### General features of the studies

This analysis was based on a total of 6 studies consisting of a total number of 1,197 participants enrolled from year 2000 to year 2024. Participants were extracted from Quasi-experimental research, cross-sectional surveys, and comparative study of opinions including operating room nurses. The countries involved included Turkey, China and the United Kingdom. The main features of the studies have been listed in Table 3.

**Table 3:** The main features of the studies.

Studies	Number of nurses in the study (*n*)	Enrollment period (year)	Country	Type of study	Type of participants
Ergin et al. (2023)	35	Year 2022	Turkey	Quasi-experimental research	Operating room nurses
Horsfall et al. (2021)	3	Year 2020	United kingdom	Cross-sectional survey	Operating room nurses
Karaarslan et al. (2024)	43	Year 2023	Western Turkey	Quasi-experimental study	Pediatrics nurses
Porto and Catal (2021)	114	Year 2018–2019	Turkey	Comparative study of opinions	Operating room nurses
Wang et al. (2024)	378	Year 2024	China	Cross-sectional study	Professional nurses including operating room nurses
Williams et al. (2024)	624	Year 2020–2021	United kingdom	Comparative study	Operating room nurses

The quality assessment based on the criteria from the NOS was given in a Supplementary material.

### Main results

The mean percentage of participants who agreed or disagreed to specific questions based on artificial intelligence were reported as results and tabulated as shown (Table 4). As per Table 4, 53.7% of the nurses understood the concept of artificial intelligence whereas 15.7% of them were not at all aware of this term and concept. 80.4% of the nurses thought that robots with artificial intelligence will benefit the nursing profession whereas 19.6% thought that these robots will not benefit the nursing profession. In addition, only 28.3% of the participants believed that robots with artificial intelligence will replace nurses whereas 71.8% of them believed that robots with artificial intelligence cannot replace nurses. Moreover, 85.9% of the nurses agreed to the fact that artificial intelligence and robotic nursing applications will reduce the workload of nurses and 14.1% believed that this will not happen. Also, 68.2% of the nurses would like to work with robot nurses in their professional life whereas 16.8% of the nurses would not like to encounter robotic nurses in their professional life. Additionally, 52.7% of the operating room nurses believed that robotic technology could help to provide a safer care whereas 21.7% of the nurses do not believe so. 60.3% of the nurses accept the application of artificial intelligence in nursing whereas 20.8% of the nurses do not accept this new concept. Moreover, 84.6% of the nurses agree that artificial intelligence will revolutionize the field of nursing whereas 25.6% do not believe so. In addition, 12.1% of the nurses think that robotic technologies are too expensive and unnecessary whereas 87.9% do not agree to this statement. Finally, 53.8% of the nurses believe that robotic checking system is time consuming whereas 46.2% believe the opposite however 77.6% nurses believe that robotic technologies are very important whereas 22.4% believe that robotic technologies are not important.

**Table 4:** The average percentage of response by Nurses to the below-mentioned questions.

	Question which were answered by the Nurses	Yes	No
1	Do you understand artificial intelligence (AI)?	53.7%	15.7%
2	Do you think robots with AI will benefit the nursing profession?	80.4%	19.6%
3	Do you think robots with AI will replace nurses?	28.3%	71.8%
4	Do you think AI and robotic nursing applications will reduce the workload of nurses?	85.9%	14.1%
5	Would you like to work with robot nurses in your professional life?	68.2%	16.8%
6	Does robotic technology help to provide a safer care?	52.7%	21.7%
7	Do you agree that application of AI in nursing can improve patients care?	60.3%	17.7%
8	Do you accept the application of AI in nursing?	79.2%	20.8%
9	Do you agree that AI will revolutionize the field of nursing?	84.6%	25.6%
10	Do you think robotic technologies are too expensive and unnecessary?	12.1%	87.9%
11	Do you think robotic checking system is time consuming?	53.8%	46.2%
12	Do you think robotic technologies are very important?	77.6%	22.4%

The above mentioned results were based on a mean percentage of the different opinions reported by the operating room nurses. However, we have been able to carry out a statistical analysis with the Revman software. Our results showed that when asked the following question: ‘Do you understand AI?’, the result was not significantly different with OR: 0.38, 95% (0.00–70.11); *p* = 0.72. Even though many operating room nurses agreed to the fact that robots with AI will benefit the nursing profession, the result was not significantly different with OR: 30.91, 95% CI: 0.55–1730.35; *p* = 0.09. The answer to the question ‘Do you think robots with AI will replace nurses?’ was also not significantly different with OR: 0.52, 95% CI: 0.05–5.72; *p* = 0.59. However, operating room nurses believe that AI and robotic nursing applications will significantly reduce the workload of nurses with OR: 75.73, 95% CI: 8.28–692.86; *p* = 0.0001. In addition, a majority of operating room nurses significantly agreed to accept the application of AI in nursing (OR: 63.70, 95% CI: 2.15–1890.57; *p* = 0.02) and majority significantly believed that AI will revolutionize in the field of nursing (OR: 15.27, 95% CI: 3.47–67.15; *p* = 0.0003). Moreover, the operating room nurses significantly disagreed to the fact that robotic technologies are too expensive and unnecessary (OR: 0.02, 95% CI: 0.01–0.04; *p* = 0.00001). In addition, the majority significantly agreed that robotic technologies are very important (OR: 12.57, 95% CI: 6.44–24.54; *p* = 0.00001). Nevertheless, even though majority of the operating room nurses agree that robotic checking system is time consuming, the result was not significant (OR: 1.38, 95% CI: 0.79–2.40; *p* = 0.26). Also, even though majority of the operating room nurses agree to the fact that the application of AI in nursing can improve patient care (OR: 13.61, 95% CI: 0.74–250.24; *p* = 0.08), and majority believe that robotic technology can help to provide a safer care (OR: 13.85, 95% CI: 0.78–245.13; *p* = 0.07), and majority of them would like to work with robotic nurses in their professional life (OR: 118.3, 95% CI: 0.76–18407.06; *p* = 0.06), the results were not statistically significant.

**Figure 2:**
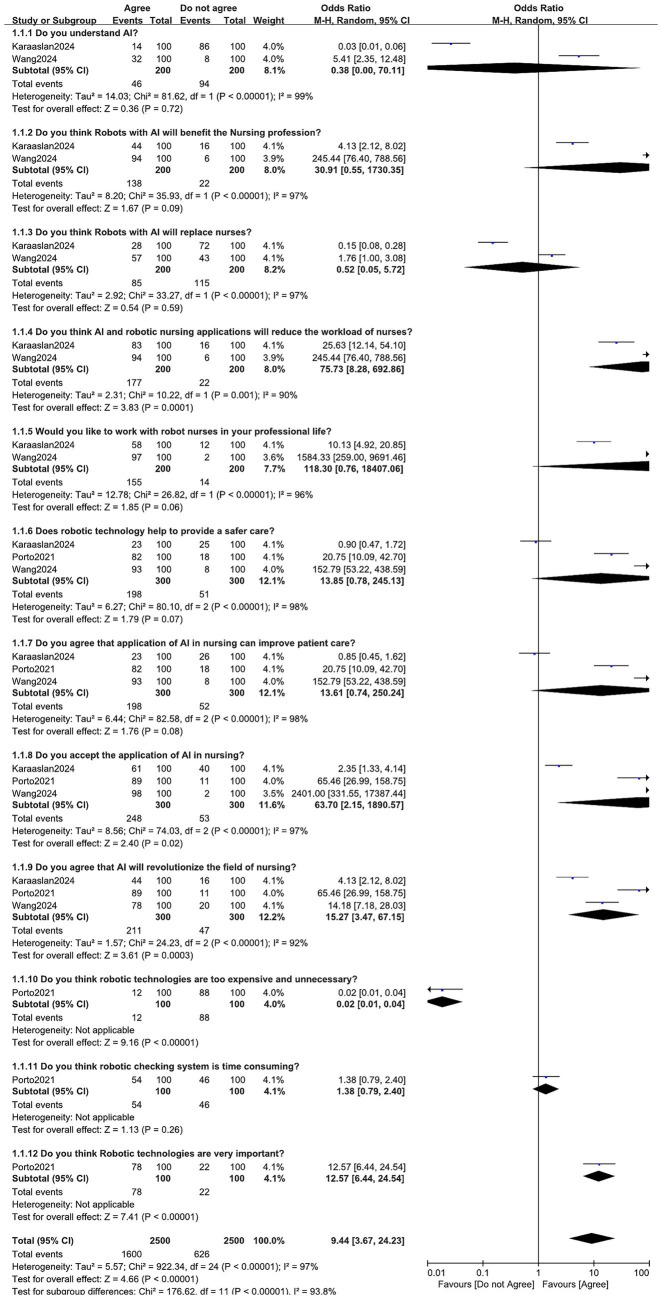
Analysis of the opinions and attitudes of the operating room nurses.

The statistical analysis has been demonstrated in Figure 2.

## Discussion

Today, big data is gaining more interests and is becoming increasingly more prevalent even in the nursing sector. Big data could impact the way nurses use to learn, practice in the hospital and conduct research and develop policy. In the future, nurses should be able to maximize the benefits of big data in order to promote human health and wellbeing. However, nurses currently lack skills required for the meaningful use of big data and therefore, the future generation of nurses should be able to improve patients’ outcomes through better quality connected health (Topaz and Pruinelli, 2017). In addition, studies have demonstrated that nurses showed varied understanding of artificial intelligence in terms of its application and its beneficial effects (Amin et al., 2025).

In this study, we focused on the opinions and attitudes of operating room nurses on artificial intelligence. Our results showed that majority of the nurses understood the concept of artificial intelligence, believed that robots with artificial intelligence will benefit the nursing profession, they believed that artificial intelligence and robotic nursing application will reduce the workload of nurses, agreed that robotic technology could help to provide a safer care, and this will revolutionize the field of nursing, and the nurses believe that these robotic technologies are very important and they accept the application of artificial intelligence in nursing. However, majority of them disagree to the fact that robots with artificial intelligence could replace nurses.

With statistical analysis, our results showed that operating room nurses believe that AI and robotic nursing applications will significantly reduce the workload of nurses. In addition, a majority of operating room nurses significantly accepted the application of AI in nursing and majority significantly believed that AI will revolutionize in the field of nursing. In addition, the majority significantly agreed that robotic technologies are very important. The operating room nurses significantly disagreed to the fact that robotic technologies are too expensive and unnecessary. Nevertheless, even though majority of the operating room nurses agree that robotic checking system is time consuming, the result was not significant.

This data analysis was based on studies that reported opinions and views of operating room nurses on the implementation of artificial intelligence in the operating room. Literature search was carried out as mentioned in the method section, and relevant studies were identified. Most of the studies which were identified reported the attitudes and opinions of operating room nurses toward artificial intelligence. Those original studies showed the percentage of nurses who agreed or disagreed to certain common questions about artificial intelligence which were asked. In this data analysis, we gathered data of questions that were similar from different studies and a mean percentage of those patients who agreed or disagreed to those questions were recorded and reported as our results. In addition, we conducted a statistical analysis wherever applicable.

Data from study Ergin et al. (2023), a single group pre and post test quasi-experimental design including 47 nurses working in the operating room were used in this current analysis. In their study, 75.4% of the participants had previously heard about the concept of artificial intelligence and robotic nursing, and over 80% of the operating room nurses believed that application of artificial intelligence in nursing would definitely reduce the workload of nurses and 77.1% believed that artificial intelligence will benefit the nursing profession.

Moreover, data from a comparative descriptive study (Porto and Catal, 2021) which was also included in our current analysis showed that majority of the nurses’ opinions about robotic technologies in the operating room was positive. Most of them believed that robotic technologies are very important and they provide a safer care to the patients. However, a minority of the nurses believed that robotic technologies are complicated and unnecessary, as also mentioned in our analysis.

In a narrative review (Andras et al., 2020), the authors stated that the use of artificial intelligence in robotic surgery could have a significant impact on future surgical training and enhance the surgical experience during a surgical procedure. The authors emphasized on the implementation of artificial intelligence in master–slave robotic surgery may allow for the careful consideration of autonomous robotic assisted surgeries. Hence, operating room nurses will have to be trained for robotic assisted surgeries. Fortunately, based on the results of this current study, more than 50% of the operating room nurses have heard of the concepts of artificial intelligence and robotic nursing, therefore, training for robotic surgery might be smoother. In addition, 68.2% of the operating room nurses would be willing to use artificial intelligence based technologies, hence, implementation of robotic assisted surgeries would mostly be welcomed by the nurses working in the operating room. Moreover, this willingness to use artificial intelligence based technologies might reduce adaptation issues following introduction of such novel technologies.

A recent study (Williams et al., 2024) which aimed to compare the ability of deep learning platform with multidisciplinary teams of the operating room in detecting cerebral aneurysms from operative videos as well as compare the ability of the neurosurgical team to detect cerebral aneurysms with and without the use of artificial intelligence as assistance, showed that artificial intelligence assisted human performance overcame both human and artificial intelligence alone. It should note that operating room nurses formed part of the team as well and in our current study, majority of the operating room nurses agreed upon the use of an artificial intelligence system in pre-operative imaging interpretation meaning that using artificial intelligence in the detection of cerebral aneurysms through operative videos was well accepted among majority of the nurses.

Our current study showed responses of operating room nurses about several questions based on artificial intelligence and its application in the operating room. In the future we believe nurses can create a novel field as clinical specialists and expand their roles as professionals (Moloney et al., 2023). The main fact is that nurses should be interested in this field of artificial intelligence for this to be possible. And we have shown that 79.2% of the operation room nurses accept the application of artificial intelligence in nursing and over 60% agree to the fact that the application of artificial intelligence in nursing will improve patients’ care and on top of that, over 80% nurses agreed to the fact that artificial intelligence will revolutionize the field of nursing.

A study based on nursing students’ perception and use of generative artificial intelligence in nursing education showed that this generative artificial intelligence was very positively welcomed in nursing education, however, guidelines would be needed for critical evaluation (Han et al., 2025). In addition, for this module to be well integrated, introductory sessions, support programs and a specific artificial intelligence friendly environment should be set up to promote artificial intelligence and prepare students for its application in nursing education. Even though studies have shown a positive attitude toward artificial intelligence in the health care sector, there is still significant gap in knowledge, skills and awareness most commonly among nurses (Vanamali et al., 2025).

The application of artificial intelligence in critical care nursing has also been studied (Porcellato et al., 2025). Artificial intelligence showed significant potential in nursing, facilitating the use of clinical practice data for research and decision making. As mentioned in our current study, most of the nurses agreed to the fact that artificial intelligence and robotic nursing applications will reduce the workload of nurses and the majority (over 80%) believed that robots with artificial intelligence will benefit the nursing profession.

Finally, though studies and research based on artificial intelligence and operating room nurses are very scarce, scientists are consistently working on new aspects to expand and carry out further research based on artificial intelligence which might rule the health sector in the near future.

This new information advances understanding in the way that opinions and attitudes of operating room nurses toward artificial intelligence and robotic surgeries were known. How do we classify operating room nurses on artificial intelligence, their knowledge, their willingness to work and assist robotic surgeries, their agreement on the benefits of artificial intelligence and robotic surgeries are important. All these points might help to understand if they would be able to adapt and if they would accept this new world where artificial intelligence will rule in the near future.

### Limitations

This study also had limitations. First of all, even though the selected studies were eligible for this analysis, not all of them reported data which could be used to represent opinions and attitudes toward artificial intelligence among operating room nurses. Another limitation could be the fact that this analysis was based on a very small population of nurses. In addition, we have included quasi-experimental studies, cross sectional surveys and studies with various populations including paediatrics nurses, and professional nurses which were not always limited to operating room nurses. Therefore, this could be another major limitation of this descriptive analysis. The inclusion of quasi-experimental studies, cross-sectional surveys, and comparative study of opinions was also a major limitation of this analysis, however, for this novel research, there were only a few such relevant studies which were published and could be used in this research work. Even though most of the studies which were included were of poor quality, we had no other option than including those studies in our analysis. Also, in study Horsfall et al. (2021), the percentage of participants was not reported. However, it was mentioned that majority of the participants agreed to certain opinions. We had no choice than include this as ‘majority of the participants agreed’ without citing the exact percentage since this was not reported in the original study. This could be another limitation of this analysis. Study Horsfall et al. (2021) consisted of a very limited number of participants (only 3 operating room nurses). However, we had to include this study because we have only a limited number of studies which were relevant to this research.

## Conclusion

In conclusion, based on the opinions and attitudes of operating room nurses toward artificial intelligence, majority of the nurses believe that artificial intelligence and robotic nursing applications will significantly reduce the workload of nurses, they believe that artificial intelligence will significantly revolutionize in the field of nursing, and they believe that robotic technologies are very important. Nevertheless, operating room nurses significantly disagree that robotic technologies are too expensive and unnecessary. However, due to the several limitations from this analysis, the results should be considered with caution.

## Data Availability

The original contributions presented in the study are included in the article/Supplementary material, further inquiries can be directed to the corresponding author.
