# Supplementary material for: Opinions and attitudes toward artificial intelligence among operating room nurses: a descriptive meta-analysis based on the comparative studies of the different opinions
Source: Front Artif Intell. 2025 Nov 19;8:1681994. doi: 10.3389/frai.2025.1681994 (PMC12672512; doi:10.3389/frai.2025.1681994)
Supplement: Supplementary file 1 [file Table_1.docx]

**Supplementary Document (S1): The methodological quality assessment of the studies using the Newcastle Ottawa Scale**

| **Studies** | **Ergin2023 [14]** | **Horsfall2020 [15]** | **Karaaslan2024 [16]** | **Porto2021 [17]** | **Wang2024 [18]** | **Williams2024 [19]** |
| --- | --- | --- | --- | --- | --- | --- |
| ***Selection*** |  |  |  |  |  |  |
| Representative of the exposed cohort | * | * | * | * | * | * |
| Selection of the external control | x | * | * | * | * | * |
| Ascertainment of exposure | x | x | x | x | x | x |
| Outcome of interest not present at the start of the study | * | * | * | * | * | * |
| ***Comparability*** |  |  |  |  |  |  |
| Main factor and additional factor based on comparability of cohorts | * | * | * | * | * | * |
| ***Outcome*** |  |  |  |  |  |  |
| Assessment of outcomes | * | * | * | * | * | * |
| Sufficient follow up time | * | * | * | * | * | * |
| Adequacy of follow up | x | x | x | x | x | x |

Abbreviations: * (present); x (absent or not reported)
